# An optimised patient information sheet did not significantly increase recruitment or retention in a falls prevention study: an embedded randomised recruitment trial

**DOI:** 10.1186/s13063-017-1797-7

**Published:** 2017-03-28

**Authors:** Sarah Cockayne, Caroline Fairhurst, Joy Adamson, Catherine Hewitt, Robin Hull, Kate Hicks, Anne-Maree Keenan, Sarah E. Lamb, Lorraine Green, Caroline McIntosh, Hylton B. Menz, Anthony C. Redmond, Sara Rodgers, David J. Torgerson, Wesley Vernon, Judith Watson, Peter Knapp, Jo Rick, Peter Bower, Sandra Eldridge, Vichithranie W. Madurasinghe, Jonathan Graffy

**Affiliations:** 1 Department of Health Sciences, York Trials Unit, University of York, York, YO10 5DD UK; 2 Podiatry Services, Harrogate and District NHS Foundation Trust, Harrogate District Hospital, Lancaster Park Road, Harrogate, UK; 3 NIHR Leeds Musculoskeletal Biomedical Research Unit, Chapel Allerton Hospital, Leeds, UK; 4 Leeds Institute of Rheumatology and Musculoskeletal Medicine, University of Leeds, Leeds, UK; 5 Nuffield Department of Orthopaedics, Rheumatology and Musculoskeletal Sciences, Kadoorie Critical Care Research Centre, John Radcliffe Hospital, University of Oxford, Oxford, UK; 6 School of Health Sciences, Áras Moyola, National University of Ireland, Galway, Ireland; 7 Lower Extremity and Gait Studies Program, Faculty of Health Sciences, La Trobe University, Bundoora, 3086 Victoria Australia; 8 The School of Human & Health Sciences, Division of Podiatry, University of Huddersfield, Huddersfield, UK; 9 Department of Health Sciences and the Hull York Medical School, University of York, York, YO10 5DD UK; 10 Medical Research Council North West Hub for Trials Methodology Research, National Institute of Health Research (NIHR) School for Primary Care Research, Manchester Academic Health Science Centre, Centre for Primary Care, University of Manchester, Oxford Road, Manchester, M13 9PL UK; 11 Pragmatic Clinical Trials Unit (PCTU), Centre for Primary Care and Public Health, Blizard Institute, Yvonne Carter Building, 58 Turner Street, London, E1 2AB UK; 12 Department of Public Health and Primary Care, University of Cambridge, Institute of Public Health, Forvie Site, Robinson Way, Cambridge, CB2 0SR UK

**Keywords:** Recruitment, Patient information, Randomised controlled trial, Retention

## Abstract

**Background:**

Randomised controlled trials are generally regarded as the ‘gold standard’ experimental design to determine the effectiveness of an intervention. Unfortunately, many trials either fail to recruit sufficient numbers of participants, or recruitment takes longer than anticipated. The current embedded trial evaluates the effectiveness of optimised patient information sheets on recruitment of participants in a falls prevention trial.

**Methods:**

A three-arm, embedded randomised methodology trial was conducted within the National Institute for Health Research-funded REducing Falls with ORthoses and a Multifaceted podiatry intervention (REFORM) cohort randomised controlled trial. Routine National Health Service podiatry patients over the age of 65 were randomised to receive either the control patient information sheet (PIS) for the host trial or one of two optimised versions, a bespoke user-tested PIS or a template-developed PIS. The primary outcome was the proportion of patients in each group who went on to be randomised to the host trial.

**Results:**

Six thousand and nine hundred patients were randomised 1:1:1 into the embedded trial. A total of 193 (2.8%) went on to be randomised into the main REFORM trial (control *n* = 62, template-developed *n* = 68; bespoke user-tested *n* = 63). Information sheet allocation did not improve recruitment to the trial (odds ratios for the three pairwise comparisons: template vs control 1.10 (95% CI 0.77–1.56, *p* = 0.60); user-tested vs control 1.01 (95% CI 0.71–1.45, *p* = 0.94); and user-tested vs template 0.92 (95% CI 0.65–1.31, *p* = 0.65)).

**Conclusions:**

This embedded methodology trial has demonstrated limited evidence as to the benefit of using optimised information materials on recruitment and retention rates in the REFORM study.

**Trial registration:**

International Standard Randomised Controlled Trials Number registry, ISRCTN68240461. Registered on 01 July 2011.

**Electronic supplementary material:**

The online version of this article (doi:10.1186/s13063-017-1797-7) contains supplementary material, which is available to authorized users.

## Background

Randomised controlled trials (RCTs) are generally regarded as the ‘gold standard’ experimental design to determine the effectiveness of an intervention. Unfortunately, many trials either fail to recruit sufficient numbers of participants, or recruitment takes longer than anticipated [1]. Such difficulties not only have an impact on the power and external validity of the study’s findings but may also increase the overall financial cost. Many trials implement methods to improve recruitment and/or retention such as the use of monetary incentives. There is, however, limited evidence as to the effectiveness of recruitment strategies in healthcare settings [2], and as a result, the UK Medical Research Council funded the Systematic Techniques for Assisting Recruitment to Trials (MRC START) research programme [3]. The aim of this programme was to “improve the evidence-base of recruitment to trials, enhance recruitment rates and make research more accessible to the public.” One key objective of the project was to develop interventions to improve recruitment to trials and test them in embedded trials within ongoing ‘host’ trials. The initial focus was on testing optimised patient information sheets and multimedia resources.

Patient information sheets (PISs) are always given to potential trial participants along with verbal information as part of the informed consent process. A trial PIS has to be reviewed and approved by an ethics committee or an internal review body; nonetheless, there remain longstanding concerns regarding their length, complexity and the level of literacy skills required to understand the information, all of which may have a negative impact on recruitment [4]. Indeed, a recent sample of 20 PISs from recently completed or ongoing RCTs were found to be lengthy (mean word count 1853, standard deviation = 960) and lacking information important to making an informed decision about trial participation [5]. However, Brierley et al. found that reducing the length of the PIS did not influence recruitment and actually yielded more ineligible patients [6]. Therefore, simply producing a shorter PIS is ineffective, and the content and interpretability of the information presented must be retained. One possible approach to improve the quality and appearance of PISs is to develop their content through a process of re-writing (for a lay audience), re-organisation, enhancing their appearance through input from a graphic designer and involving user testing [7, 8]. The use of a professionally designed information pack was seen to have a small positive effect on initial response rates relative to a ‘standard’ pack (2.7% difference in response rates, 95% confidence interval (CI) −0.06 to 5.5%, *p* = 0.06) in an RCT embedded in an Avon Longitudinal Study of Parents and Children (ALSPAC) cohort [9]. However, undertaking commercial user testing and incorporating graphic design input into PISs is costly, especially if the process needs to be completed for each trial; therefore, it has been suggested that a ‘bank’ of template PISs could be developed (which have been re-written and have undergone bespoke user testing and graphic design) in different populations. Researchers could select the most appropriate template on which to base their trial PIS according to their particular study population.

Embedding recruitment trials into ongoing trials is an efficient method to test recruitment strategies, and to help fill the knowledge gap with minimal resource use [10]. Table 1 presents a Consolidated Standards of Reporting Trials (CONSORT) checklist for reporting embedded recruitment trials. The REFORM study team were invited by the MRC START programme to embed a recruitment methodology trial within the REducing Falls with ORthoses and a Multifaceted podiatry intervention (REFORM) study. The REFORM study was selected, as the aims of the MRC START programme were compatible with both the need to maximise recruitment to the REFORM trial and the ethos of the York Trials Unit (YTU) to develop an understanding of effective recruitment interventions. The aim of the designed three-arm embedded trial was to determine if the number of participants recruited, randomised and retained to the REFORM trial could be improved by the use of both a bespoke user-tested and a template-developed optimised PIS.

**Table 1:** Checklist of items for reporting embedded recruitment trials

Section/topic and item no.	CONSORT 2010 (standard) checklist item	Extension for embedded recruitment trials
Title and abstract		
1a	Identification as a randomised trial in the title	Identification as an *embedded randomised recruitment trial* in the title
1b	Structured summary of trial design, methods, results and conclusions (for specific guidance see CONSORT for abstracts)	Structured summary of *embedded recruitment trial* design, methods, results and conclusions (for specific guidance see CONSORT for abstracts)
Introduction		
Background and objectives		
2a	Scientific background and explanation of rationale	Scientific background and explanation of rationale *for embedded recruitment trial including a brief description of the host trial(s) as appropriate*
2b	Specific objectives or hypotheses	Specific objectives or hypotheses *for embedded recruitment trial*
Methods		
Trial design		
3a	Description of trial design (such as parallel, factorial) including allocation ratio	Description of *embedded recruitment trial* design (such as parallel, factorial, *cluster*) including allocation ratio
3b	Important changes to methods after trial commencement (such as eligibility criteria), with reasons	Important changes to methods *of the embedded recruitment trial* after commencement (such as eligibility criteria), with reasons
Participants		
4a	Eligibility criteria for participants	Eligibility criteria for participants *for embedded recruitment trial, including any differences from those for the host trial(s)*
4b	Settings and locations where the data were collected	Settings and locations where the *embedded recruitment trial was carried out, including a brief description of the host trial(s) as appropriate*
Interventions		
5	The interventions for each group with sufficient details to allow replication, including how and when they were actually administered	The interventions for each group *(including control group) within the embedded recruitment trial* with sufficient details to allow replication, including how, *where* and when they were actually administered
Outcomes		
6a	Completely defined pre-specified primary and secondary outcome measures, including how and when they were assessed	Completely defined pre-specified primary and secondary outcome measures for the embedded recruitment trial, including how and when they were assessed
6b	Any changes to trial outcomes after the trial commenced, with reasons	Any changes to embedded recruitment trial outcomes after the embedded recruitment trial commenced, with reasons
Sample size		
7a	How sample size was determined	How sample size for embedded recruitment trial was determined
7b	When applicable, explanation of any interim analyses and stopping guidelines	When applicable, explanation of any interim analyses and stopping guidelines for embedded recruitment trial
Randomisation		
Sequence generation		
8a	Method used to generate the random allocation sequence	Method used to generate the random allocation sequence *for embedded recruitment trial*
8b	Type of randomisation; details of any restriction (such as blocking and block size)	Type of randomisation; details of any restriction (such as blocking and block size) *in embedded recruitment trial*
Allocation concealment mechanism		
9	Mechanism used to implement the random allocation sequence (such as sequentially numbered containers), describing any steps taken to conceal the sequence until interventions were assigned	Mechanism used *in the embedded recruitment trial* to implement the random allocation sequence (such as sequentially numbered containers), describing any steps taken to conceal the sequence until interventions were assigned
Implementation		
10	Who generated the random allocation sequence, who enrolled participants and who assigned participants to interventions	Who generated the random allocation sequence*(s)*, who enrolled participants and who assigned participants to *embedded recruitment* interventions
Blinding		
11a	If done, who was blinded after assignment to interventions (for example, participants, care providers, those assessing outcomes) and how	If done, who was blinded after assignment to *embedded recruitment* interventions (for example, participants, care providers, those assessing outcomes) and how
11b	If relevant, description of the similarity of interventions	If relevant, description of the similarity of interventions *in the embedded recruitment trial*
Statistical methods		
12a	Statistical methods used to compare groups for primary and secondary outcomes	Statistical methods used to compare groups for primary and secondary outcomes *of the embedded recruitment trial*
12b	Methods for additional analyses, such as subgroup analyses and adjusted analyses	Methods for additional analyses, such as subgroup analyses and adjusted analyses *for embedded recruitment trial*
Results		
Participant flow (a diagram is strongly recommended)		
13a	For each group, the numbers of participants who were randomly assigned, received intended treatment and were analysed for the primary outcome	For each group *in the embedded recruitment trial,* the numbers of participants who were randomly assigned, received intended treatment and were analysed for the primary outcome
13b	For each group, losses and exclusions after randomisation, together with reasons	For each group, losses and exclusions after randomisation *to embedded recruitment trial,* together with reasons
Recruitment		
14a	Dates defining the periods of recruitment and follow-up	Dates defining the periods of recruitment and follow-up *for both embedded recruitment trial and host trial(s)*
14b	Why the trial ended or was stopped	Why the *embedded recruitment trial* ended or was stopped
Baseline data		
15	A table showing baseline demographic and clinical characteristics for each group	*If possible* a table showing baseline characteristics *of each arm of the embedded recruitment trial*
Numbers analysed		
16	For each group, number of participants (denominator) included in each analysis and whether the analysis was by original assigned groups	For each group *in the embedded recruitment trial,* number of participants (denominator) included in each analysis and whether the analysis was by original assigned groups
Outcomes and estimation		
17a	For each primary and secondary outcome, results for each group, and the estimated effect size and its precision (such as 95% confidence interval)	For each primary and secondary outcome, results for each group *in the embedded recruitment trial,* and the estimated effect size and its precision (such as 95% confidence interval)
17b	For binary outcomes, presentation of both absolute and relative effect sizes is recommended	For binary outcomes *in the embedded recruitment trial*, presentation of both absolute and relative effect sizes is recommended
Ancillary analyses		
18	Results of any other analyses performed, including subgroup analyses and adjusted analyses, distinguishing pre-specified from exploratory	Results of any other analyses performed *for embedded recruitment trial*, including subgroup analyses and adjusted analyses, distinguishing pre-specified from exploratory
Harms		
19	All important harms or unintended effects in each group (for specific guidance see CONSORT for harms)	All important harms or unintended effects in each group *for both the embedded recruitment trial and host trial(s)* (for specific guidance see CONSORT for harms)
Discussion		
Limitations		
20	Trial limitations, addressing sources of potential bias, imprecision and, if relevant, multiplicity of analyses	*Embedded recruitment trial* limitations, addressing sources of potential bias, imprecision and, if relevant, multiplicity of analyses
Generalisability		
21	Generalisability (external validity, applicability) of the trial findings	Generalisability (external validity, applicability) of the *embedded recruitment trial* findings
Interpretation		
22	Interpretation consistent with results, balancing benefits and harms, and considering other relevant evidence	Interpretation consistent with results *of the embedded recruitment trial,* balancing benefits and harms, and considering other relevant evidence
Other information		
Registration		
23	Registration number and name of trial registry	Registration number and name of trial registry *(for all host trials and embedded recruitment trial if available)*
Protocol		
24	Where the full trial protocol can be accessed, if available	Where the *embedded recruitment trial* protocol can be accessed, if available
Funding		
25	Sources of funding and other support (such as supply of drugs), role of funders	*For embedded recruitment trial,* sources of funding and other support, role of funders and collaborators

## Methods

### Ethics approval

This trial was embedded within the National Institute for Health Research (NIHR)-funded REFORM study [11], which aims to evaluate the clinical and cost effectiveness of a podiatry intervention for the prevention of falls in older people. Ethical approval for the REFORM study was given by NRES East of England – Cambridge East Research Ethics Committee and the University of York, Department of Health Sciences Research Governance Committee. Ethical approval for the PIS embedded methodology trial was given via a substantial amendment to the same committees. The embedded methodology trial is registered as a substudy to REFORM (ISRCTN68240461).

### Participant recruitment

REFORM is a cohort RCT [12] in which patients were initially recruited to an observational cohort before potentially being randomised into an RCT. Electronic medical notes at participating National Health Service (NHS) podiatry clinics were searched to identify community-dwelling patients eligible for the REFORM observational cohort (i.e. those who were over the age of 65 and had attended a routine podiatry appointment within the past 6 months). Potentially eligible participants were mailed an invitation pack (letter of invitation, one of the three PISs being evaluated in this embedded trial, consent form, screening questionnaire and prepaid envelope) asking whether they would like to participate in the REFORM study. The information and consent process covered all aspects of the cohort and trial, and so only one PIS was seen by participants whether they went on to be randomised into the REFORM trial or not. Participants who returned their completed consent and screening form to the YTU by post were assessed by researchers at the YTU for eligibility. Participants were ineligible if they reported neuropathy, dementia or another neurological condition such as Parkinson’s or Alzheimer’s disease; were unable to walk household distances without the help of a walking aid; or had a lower limb amputation or were unwilling to attend their local podiatry clinic. Eligible participants were sent a baseline questionnaire and monthly falls calendars. Participants who returned a baseline questionnaire and at least one monthly falls calendar (recording falls in the previous month) were included in the observational cohort. Those cohort participants who had either had one fall in the past 12 months or one fall in the past 24 months requiring hospital attention, or who reported a fear of falling on the baseline questionnaire (worried about falling at least some of the time in the past 4 weeks) were then eligible for randomisation to the REFORM trial. During the course of the trial, cohort participants who went on to have a fall could then become eligible for the REFORM trial. In order to minimise the delay between randomisation and the podiatrist seeing the participants in clinic for their REFORM appointment, participants were randomised when clinics had capacity and not at the point of becoming eligible for the trial.

At the point at which this embedded trial was nested within the host trial, the next podiatry clinics due to begin recruitment were Harrogate, Leeds, Scarborough, Selby and Sheffield. All patients due to be sent a REFORM invitation pack from these clinics were randomised to one of the three arms in the embedded methodology trial which determined which format of PIS they were sent.

### Randomisation and blinding

This was an embedded, individually randomised, recruitment trial. A list of patients was generated and ordered by NHS number. Each patient was assigned a unique identification number. An independent data manager at the YTU, who was not involved in the recruitment of participants, generated the allocation sequence for the embedded methodology trial electronically. Randomisation was stratified by centre, using a single large block per site, which corresponded to its total sample size. Participants were allocated 1:1:1 to receive, in their invitation pack, either (1) the control PIS and control invitation letter; or (2) an optimised version of the PIS and invitation letter developed through bespoke user testing; or (3) an optimised template-developed PIS and the original invitation letter. Patients were then sent the allocated invitation pack by members of the research team based at the University of York. The researchers, patients and podiatrists were blind to the allocation. Patients who received the information sheet were unaware that they had been randomised to receive different information leaflets.

### Control group

The ‘control’ PIS was the original PIS developed for the REFORM trial. It was written in accordance with the standard National Research Ethics Service template available at the time at which the study was set up. It underwent several revisions by the research team and was five pages of A4 paper in length. The invitation letter was one A4 page on NHS trust headed paper. The control PIS is shown in Additional file 1.

### Intervention group: ‘bespoke user-tested’ PIS

The ‘bespoke user-tested’ PIS was developed in the following way: it was re-written for a lay audience (led by author PK), re-organised into eight subsections, had graphic design input and underwent user testing. The graphic design and user testing were both undertaken by commercial companies. Staff members who had considerable experience in writing patient material and a graphic designer revised the content and layout of the control PIS. A contents list was placed on the front page along with a list of key points about the study, with the main text divided into eight sections. Sentences and paragraphs were shortened and bullet points used for lists. The PIS then underwent three rounds of user testing, each involving 10 members of the public with limited podiatry- or trial-specific knowledge. During the user-testing process, the users (a group demographically similar to the REFORM trial target population but with no history of falls) were asked to find key pieces of information in the PIS relating to: the nature and purpose of the trial; the process and meaning of consent; and trial procedures and safety issues. Questions were arranged in such a way to ensure they did not match the order within the PIS. The final version of the PIS was printed on one sheet of double-sided A3 paper and folded to form a booklet. The control one-page invitation letter was revised and underwent user testing alongside the PIS, resulting in shortened text and the addition of bullet points to clarify what to do if the participant wished to take part. The user-tested PIS is shown in Additional file 2.

### Intervention group: ‘template-developed’ PIS

The ‘template-developed’ PIS was written by three research fellows with more than 12 years’ experience of recruiting patients to a range of randomised trials and who were currently working on the REFORM study. The content, layout and style were revised using a previously bespoke user-tested PIS designed for an earlier trial conducted in a similar aged population [13]. In this process, the control PIS text was shortened and divided into seven sections, a contents list included and bullet points added to clarify what the participant should do if he/she wished to take part. A green colour scheme was used to match the trial logo. The PIS was reviewed and revised further by two other experienced researchers. The Public and Patient Involvement (PPI) group reviewed this PIS and gave feedback about its readability, layout, font size and content; however, no graphic design input or formal user testing was undertaken. The revised version was printed on one sheet of double-sided A3 paper and folded to form a booklet. Participants in the template-developed PIS group were sent the same patient invitation letter as the control group. The template-developed PIS is shown in Additional file 3.

### Primary outcome

The primary outcome was the proportion of patients in each group who went on to be randomised into the REFORM trial.

### Secondary outcomes

Secondary outcomes were as follows:

- Proportion of patients in each group who were recruited into the REFORM cohort
- Proportion of patients retained in the trial at 3 months post randomisation defined as returning at least the first 3 months’ worth of falls calendars from the date of randomisation.

### Sample size

As is usual with an embedded trial within a trial, no formal power calculation was undertaken for this embedded methodology trial, but the MRC START programme set out to include trials which planned to approach enough potential participants to allow 400 potential recruits to receive each recruitment intervention. Five centres were involved in the substudy; thus, the sample was constrained to only include participants eligible for the REFORM study who were due to be mailed a recruitment pack from these centres.

### Statistical analysis

The proportion of participants who: returned a consent form; were recruited into the cohort; were randomised into the main trial; and were retained in the trial is presented for the three groups. Odds ratios (ORs) and 95% confidence intervals (CIs) for each of the three pairwise comparisons were obtained from mixed logistic regression models adjusting for PIS allocation as a fixed effect and trial centre as a random effect. Analysis was conducted in Stata v13, using two-sided tests at the 5% significance level and based on the principles of intention to treat.

To account for the participants in the embedded trial of the newsletter who overlapped with participants in this trial, sensitivity analyses were conducted including a covariate for receiving a newsletter in all logistic regressions.

## Results

**Fig. 1:**
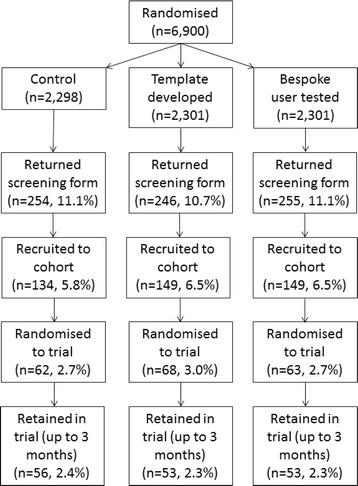
Flow diagram to depict the flow of participants in the PIS embedded methodology trial

A total of 6900 recruitment packs were sent to potential REFORM study participants across five centres between March 2013 and May 2014: Harrogate (*n* = 500, 7.3%), Scarborough (*n* = 1000, 14.5%), Selby (*n* = 1000, 14.5%), Sheffield (*n* = 1600, 23.2%) and Leeds (*n* = 2800, 40.6%) (Fig. 1). Potential participants were sent a recruitment pack containing either the control version of the PIS (*n* = 2298, 33.3%), the template-developed PIS (*n* = 2301, 33.4%) or the user-tested PIS (*n* = 2301, 33.4%).

### Responses to invitation

A consent form and screening questionnaire were returned by 755 or 10.9% of individuals (control *n* = 254 (11.1%); template-developed *n* = 246 (10.7%); user-tested *n* = 255 (11.1%)). PIS allocation did not significantly improve the response (chi-square 0.22, degrees of freedom = 2, *p* = 0.89). No one group was more likely to respond (template-developed vs control OR 0.96, 95% CI 0.80–1.16, *p* = 0.69; bespoke user-tested vs control OR 1.00, 95% CI 0.83–1.21, *p* = 0.98; and bespoke user-tested vs template-developed OR 1.04, 95% CI 0.86–1.25, *p* = 0.67).

### Randomised to REFORM trial

Eligible, consenting participants were then sent a baseline questionnaire (control *n* = 145 (6.3%); template-developed *n* = 158 (6.9%); bespoke user-tested *n* = 160 (7.0%)). A baseline form and at least one falls calendar were returned by 134 (5.8%) individuals in the control group, 149 (6.5%) in the template-developed group and 149 (6.5%) in the bespoke user-tested group. These participants were recruited to the cohort of potentially eligible participants.

Of these cohort patients, 161 were immediately eligible for randomisation into REFORM, and of these, 122 could be randomised as there was sufficient capacity in the clinics to see them. A further 71 patients in the cohort became eligible for the trial as they subsequently reported a fall and were able to be seen in clinic. Therefore, a total of 193 of the 6900 potential participants (2.8%) went on to be randomised into the main REFORM trial (control *n* = 62 (2.7%, 95% CI 2.0–3.4%); template-developed *n* = 68 (3.0%, 95% CI 2.3–3.6%); bespoke user-tested *n* = 63 (2.7%, 95% CI 2.1–3.4%)). The difference in percentages and their 95% CIs are as follows: for template-developed vs controls there was a 0.3 percentage point difference (95% CI −0.7 to 1.2, *p* = 0.60); bespoke user-tested vs control 0.0 (95% CI −0.9 to 1.0, *p* = 0.93); and bespoke user-tested vs template-developed −0.2 (95% CI −1.2 to 0.7, *p* = 0.66).

Descriptive data for these participants are presented in Table 2. PIS allocation did not improve recruitment to the trial (chi-square 0.33, degrees of freedom = 2, *p* = 0.85). The odds ratios for the three pairwise comparisons were: template-developed vs control 1.10 (95% CI 0.77–1.56, *p* = 0.60); bespoke user-tested vs control 1.01 (95% CI 0.71–1.45, *p* = 0.94); and bespoke user-tested vs template-developed 0.92 (95% CI 0.65–1.31, *p* = 0.65).

**Table 2:** Characteristics of participants randomised into the main trial by PIS allocation

Characteristic	Control (*n* = 62)	Template-developed (*n* = 68)	Bespoke user-tested (*n* = 63)	Total (*n* = 193)
Gender, *n* (%)				
Male	24 (38.7)	32 (47.8)	27 (43.6)	83 (43.5)
Age				
Mean (SD)	78.3 (5.8)	78.6 (6.6)	77.5 (7.8)	78.1 (6.8)
Fallen in previous 6 months? *n* (%)				
Yes	23 (37.1)	29 (43.3)	27 (42.9)	79 (41.2)
No	39 (62.9)	36 (53.7)	36 (57.1)	111 (57.8)
Don’t know	0 (0.0)	2 (3.0)	0 (0.0)	2 (1.0)
If fallen in previous 6 months, how many times?				
Median (min,max)	1 (1, 5)	1 (1, 20)	1 (1, 6)	1 (1, 20)
Worried about having a fall during the previous 4 weeks				
All of the time	4 (6.5)	3 (4.5)	4 (6.4)	11 (5.7)
Most of the time	1 (1.6)	4 (6.0)	2 (3.2)	7 (3.7)
A good bit of the time	6 (9.7)	3 (4.5)	4 (6.4)	13 (6.8)
Some of the time	19 (30.7)	13 (19.4)	9 (14.3)	41 (21.4)
A little of the time	23 (37.1)	27 (40.3)	29 (46.0)	79 (41.2)
None of the time	9 (14.5)	17 (25.4)	15 (23.8)	41 (21.4)
Short Falls Efficacy Scale – International (FES-I)^a^				
Mean (SD)	13.1 (5.0)	11.7 (4.3)	12.1 (4.5)	12.3 (4.7)
Frenchay Activities Index (FAI)^a^				
Mean (SD)	47.2 (6.7)	46.5 (7.2)	46.4 (7.1)	46.6 (7.0)

^a^FES-I scored from 7–28, higher score indicates greater concern about the possibility of falling; FAI scored from 15–60, higher score indicates greater activity

### Retention rates

In total, 162 participants were retained in the trial (control *n* = 56 (2.4%); template-developed *n* = 53 (2.3%); user-tested *n* = 53 (2.3%)).

### Sensitivity analyses

Three hundred and thirty four participants also received a newsletter in their recruitment pack (control *n* = 120 (5.2%); template-developed *n* = 112 (4.9%); bespoke user-tested *n* = 102 (4.4%)). In the sensitivity analyses, only a negligible difference in the estimates, their associated 95% CIs and *p* values was observed.

## Discussion

We have evaluated the effectiveness of two optimised patient information sheets (PISs) relative to the standard control version developed using the NRES template PIS to increase recruitment to the REFORM trial. Previous studies investigating the method of delivering paper-based information to patients have found that reducing the length of the PIS or supplementing the PIS with a booklet on clinical trials has little or no impact on trial recruitment, but that the use of a professionally designed information pack may be effective [6, 9, 14]. Alternative strategies could be evaluated in future embedded trials. Bower et al. [10] have identified three areas which merit further investigation. These include training site staff, communication with patients and incentives. The key finding of this study is that the use of enhanced PISs did not significantly increase recruitment or retention. The overall recruitment rate to the REFORM trial (2.8%) and the REFORM cohort (6.3%) was low, so it may be that there was insufficient power to detect a difference. The MRC START group will undertake a meta-analysis of six embedded trials evaluating enhanced information sheets which will have more power to provide evidence of effectiveness. Small differences in recruitment rates could make a large difference to trials similar to REFORM, which required a sample size of nearly 900 participants. For example, a 3% uptake would require 30,000 invitations, but if uptake to the study increased to 4%, then 7500 fewer recruitment packs would be required, saving up to £35,000 in packing and postage costs alone. This would be a significant saving, even after incorporating the cost of professional graphic design and bespoke user testing. In this study, there were no statistically significant differences between the bespoke user-tested and template-developed information sheets, so it may be beneficial to develop a bank of template information sheets rather than each trial having to take account of the additional time and expense of professional user testing. Alternatively, since many trials include PPI, a more structured approach to reviewing and developing the information sheet could be evaluated and then, if effective, adopted within PPI groups.

One reason why there may have been little evidence of a difference is that the ‘standard’ PIS has been developed by experienced researchers, and consequently it may be difficult to achieve a significantly improved information sheet. It could also be that the optimised PISs increased the interpretably and clarity of the provided information, which deterred some patients from responding. Unlike the other host trials in the START programme, REFORM used a cohort randomised controlled trial design. Participants were recruited first to the observational cohort, and then eligible participants were randomised to the REFORM trial. Therefore, optimised PISs were more likely to have an effect on recruitment to the observational cohort than randomisation into the REFORM trial. As there was a lack of effect on recruitment to the observational cohort, it was unlikely that there would be an impact on recruitment or retention in the trial.

There are some potential limitations to this study. First, the results of this study are applicable only to those participants over the age of 65 years attending routine podiatry clinics. Further studies should substantiate the study results in other populations. Second, due to the nature of the intervention, it was not possible to blind administrative staff who mailed out the recruitment packs, but it is unlikely that allocation subversion could have taken place. Whilst participants were aware of which PIS they received, they were unaware that an embedded trial was being conducted and that recruitment to the REFORM study was being monitored. Finally, it was not possible to evaluate whether the enhanced information sheets affected the speed of recruitment, as podiatry clinics were limited to the number of participants they could see at any one time.

## Conclusions

Optimised participant information sheets are one of a number of recruitment interventions amenable to testing through embedded trial methodology. Whilst the results of this study suggest limited benefits to enhancing patient information sheets, the findings add to the body of evidence around the effectiveness of recruitment strategies and may potentially help save time and money in future trials. The results of this study and the other embedded studies for the MRC START programme are to be incorporated into a meta-analysis which will provide more robust evidence as to the effectiveness of these types of recruitment strategies.

### Guidelines for reporting embedded recruitment trials

The guidelines are obtained from the following source: Vichithranie W. Madurasinghe and Sandra Eldridge on behalf of the MRC START group and Gordon Forbes on behalf of the START Expert Consensus group. *Trials*. 2016;17:27. Published online 2016 Jan 14. doi: 10.1186/s13063-015-1126-y.

## Additional files

Additional file 1: Original, control patient information sheet. (DOC 103 kb)

Additional file 2: Bespoke user-tested patient information sheet. (PDF 328 kb)

Additional file 3: Template-developed patient information sheet. (PDF 162 kb)

## Abbreviations

ALSPAC: Avon Longitudinal Study of Parents and Children
CI: Confidence interval
ISRCTN: International Standard Randomised Controlled Trial Number
MRC START: Medical Research Council Systematic Techniques for Assisting Recruitment to Trials
NHS: National Health Service
NRES: National Research Ethics Service
OR: Odds ratio
PIS: Patient information sheet
PPI: Public and Patient Involvement
RCT: Randomised controlled trial
REFORM: REducing Falls with ORthoses and a Multifaceted podiatry intervention
YTU: York Trials Unit
