# Supplementary material for: An optimised patient information sheet did not significantly increase recruitment or retention in a falls prevention study: an embedded randomised recruitment trial
Source: Trials. 2017 Mar 28;18:144. doi: 10.1186/s13063-017-1797-7 (PMC5370466; doi:10.1186/s13063-017-1797-7)
Supplement: Supplementary file 1 — Original, control patient information sheet. (DOC 103 kb) [file 13063_2017_1797_MOESM1_ESM.doc]

**Your invitation to participate in a research study.**

**Can you help?**

We would like to invite you to take part in our research study. Before you decide whether to take part, it is important for you to understand why the study is being done and what it will involve. Please take time to read this information sheet carefully and discuss it with your family or friends if you wish. Ask us if there is anything that is not clear, if you would like more information or if you would like help with completing the forms – our contact details are given at the end of this leaflet.

**What is the purpose of the study?**

As you know, falling is a common problem among people over the age of 65 years. It has been estimated that up to half of people aged over 80 fall each year. People may fall for a variety of reasons, including problems with balance or due to poor vision. Unfortunately, some of these falls will cause serious injury, such as a broken bone.

People often think that falls are an unavoidable result of getting older and that little can be done to stop them. It may not be possible to prevent falls completely, however, there are many different ways to help reduce the number of falls someone has for example undertaking exercises, checking your eyesight and reviewing your medication.

In this study we are looking at the health of people over the age of 65. We are also interested in finding out the likelihood of people falling and ways of reducing the number of falls older people have. For example we would like to know if foot and ankle exercises or wearing insoles in your shoes prevent falls.

**Why have I been approached?**

People aged over 65 , who have attended the [insert podiatry clinic] clinic are being sent information about the study and an invitation to take part in the study.

Because this information has been sent to you by the podiatry clinic your name and address is not available to the researchers unless you choose to give it to them by agreeing to take part in the study.

We hope about 1,700 people will agree to take part in this study from across the UK.

**Do I have to take part?**

No, it is entirely up to you whether or not you decide to take part. Participation in the trial is entirely voluntary. If you decide to take part, you are still free to withdraw at any time, without giving a reason. A decision to withdraw at any time, or a decision not take part, will not affect the standard of care you receive. If you would like more information then please feel free to contact us, our details are at the end of this leaflet.

If you want to take part please keep this information sheet. You will be asked to sign a consent form, a copy will be returned to you to keep.

**Expenses and payments**

Unfortunately, we are not able to offer any direct expenses or payments to patients who participate in the study.

**What will be involved if I agree to take part?**

This study will take us three and a half years to complete but if you agree to take part in the study we will only ask for your help for a maximum of 24 months. If you agree to take part in the study you will be asked to complete some questionnaires about yourself and return them to us in a prepaid envelope. We will then send you a falls calendar, which we will ask you to complete and send back to us each month. We will also send another set of questionnaires to you six months later and then after 12 months for you to complete. We expect it will take you about 20 minutes to compete each of the questionnaires we send you. If you do have a fall, we will ask you to ring the research team at the University of York, who will ask you some questions about your fall. The information you provide will allow us to follow your health and to see if it changes over time. It will also contribute to the wider study we are carrying out about the health of people over 65 and will let us look at the benefit of long term podiatry treatments.

If you take part in the study your GP care and care you received from the podiatrist will continue as usual. You will receive a patient information sheet giving advice about how to reduce the possibility of you having a fall. Some people may be offered some extra podiatry visits at a later date to look at ways of reducing falls. It will not be possible to offer everyone some extra podiatry visits, so these people will be selected according to the play of chance (randomly), like using the toss of a coin.

In addition, we may contact you to ask if you would like to take part in an interview with one of our researchers to discuss your views about how we can improve balance and reduce the number of falls people have and about taking part in the study.

The study results will be written up and published, a summary of which will be made available to you.

**What will happen if I agree to take part in the study?**

We want to know if improved podiatry care can reduce falls. If you are interested in having extra podiatry care, including foot and ankle exercises then you may be offered this as part of the study at a later date.

**What will happen if I am offered the podiatrist package of care?**

If you are offered the extra podiatry care you will be asked to see a podiatrist at your podiatry clinic on two occasions. The first appointment will last for about an hour and the second appointment for about 20 minutes. Your podiatrist will contact you to make appointments which are convenient for you.

At the first appointment the podiatrist will assess your outdoor shoes, give you advice about your footwear and undertake routine podiatry care as required. They will then measure your feet and fit an orthotic device – a type of insole which is worn in your shoe. You will then be shown some foot and ankle exercises and asked to do these at home three times a week. You will be given an exercise diary and asked to complete this and send it back to the University of York during the course of the trial. You will also receive a patient information sheet giving advice about how to reduce the possibility of you having a fall. At your second appointment, the podiatrist will check the fitting of your orthotic device and check how you’re getting on with the foot and ankle exercises.

**What will happen if I am not offered extra podiatry visits?**

If you are not offered extra podiatry care, your GP care will continue as usual. If you are already seeing a podiatrist for treatment which is not related to the trial, you will continue to see them as usual. If you are not currently receiving treatment from a podiatrist, you will not be invited to attend the podiatry clinic. You will receive a patient information sheet giving advice about how to reduce the possibility of you having a fall.

We would still ask you to complete and return the questionnaires mentioned above.

**What are the possible benefits of taking part in this study?**

We cannot promise that taking part in this study will help you, but the information we get from the study may help us to find out how we can help improve balance and reduce the number of falls people have.

**What are the possible disadvantages of taking part in this study?**

Taking part in this study will involve some of your time to complete questionnaires. If you are randomly allocated to receive the podiatry package of care, you will be contacted by a podiatrist and asked to attend the podiatry clinic. We cannot think of any other disadvantages.

**Will the information in the study be confidential?**

Any information you provide us with will be treated in confidence. At the beginning of the study we will record your name, address, telephone number and date of birth and keep a copy of your signed consent form. This information will be stored securely at the University of York in accordance with the Data Protection Act 1998. Your name will not be mentioned in any publications arising from the study and we will ensure that individuals cannot be identified from details in reports of the study results. If you withdraw from the study at any time, unless you inform us otherwise, the information you have already provided will be used in anonymous form for the purposes of the study.

If you consent to take part in the research, members of the REFORM research team from the University of York (for the purposes of checking data collection) may inspect your medical records. People from regulatory authorities such as the NHS Trust where the study is being conducted may also look at your records to check that the study is being carried out correctly.

**Will I be approached about taking part in any other studies?**

If you agree to take part in this research, if you consent you may be invited to join other research studies about how to improve balance and reduce the number of falls being carried out by researchers in the REFORM team. You do not have to take part in any related studies, and you will be sent more information about them before you decide. If you do agree to us contacting you about other studies we will keep all personal and anonymised data for a total of 5 years to allow us to do this.

**Will my GP be involved?**

If you consent we will inform your GP if you agree to participate in this research. If you consent we will also contact your GP if we have any concerns about your health during your participation. If you do have a fall, if you agree we may contact your GP to find out some further information.

**What if there is a problem?**

If you have a concern about any aspect of this study, you should ask to speak to the trial co-ordinator who will do their best to answer your questions, their details are at the end of this leaflet. If you do not want to speak to the trial co-ordinator you can contact the local principal investigator ([PI name and contact telephone]) or your local Patient Advice and Liaison Service (PAL). [Insert site specific PALS details here.]

While we anticipate no harm or distress to anyone as a result of this study it is important to state that there are no special compensation arrangements. If you are harmed due to someone’s negligence, then you have ground for legal action but you may have to pay for it. Regardless of this if you wish to complain, or have any concerns about any aspect of the way you have been approached or treated during the course of this study, the normal National Health Service complaints mechanisms are available to you.

**Yes, I would like to take part in the study – what do I need to do now?**

Please complete and sign the enclosed yellow consent form and background information form and return them in the prepaid envelope provided. If you need any help with completing the forms, please phone us and we’ll be happy to help. We will write to you again in a few weeks time to ask you to complete some simple questionnaires. If you have decided to participate in the study, if you agree we will let you and your GP know how you are involved in the study.

**I’m not sure about taking part – where can I get more information about the study?**

We would be very pleased to answer any questions you may have. Please contact either the study coordinator [insert name and telephone number] or your local podiatrist [Name of local podiatrist contact at podiatry clinic delivering intervention], on [telephone number].

**Is there anyone else I can talk to about the study?**

For further general information and advice about taking part in research, you can contact the Patient Advice and Liaison Service (PAL). [Insert site specific PALS details here.]

**How can I find out about the results of the study?**

This study is due to finish in summer 2015. All patients who have consented to take part in the research will be sent a summary of the results. Participants can request copies of any published data by contacting the study coordinator.

If you decide not to take part in the study but would like to receive a copy of the results you can contact the York Trials Unit directly. Our contact details are at the end of this leaflet.

**Who is involved in organising and funding this study?**

This study is being organised by the University of York. The research has been funded by the Department of Health, National Institute of Health Research Health Technology Assessment programme. All research in the NHS is looked at by an independent group of people, called a Research Ethics Committee, to protect your safety, rights, wellbeing and dignity. This research has been reviewed and approved by [insert name of Ethics committee] Research Ethics Committee.

**Thank you for reading this information sheet**

**If you require any further information please contact us.** A friend or relative may speak to us on your behalf if you wish. There is an answering machine available 24 hours a day, so please leave a message and one of the research team will contact you as soon as possible.

The REFORM study also has a website at [insert web address]

**Contact details:-**

**Study coordinator:** Sarah Cockayne

**Tel:** 01904 321736

**Address:** York Trials Unit, Lower Ground Floor, ARRC Building, University of York, Heslington, York, YO10 5DD

**Podiatrist contact**: [Insert name of podiatrist]

**Tel:** [Insert telephone number]

**Address:** [Insert address]
