# Supplementary material for: An optimised patient information sheet did not significantly increase recruitment or retention in a falls prevention study: an embedded randomised recruitment trial
Source: Trials. 2017 Mar 28;18:144. doi: 10.1186/s13063-017-1797-7 (PMC5370466; doi:10.1186/s13063-017-1797-7)
Supplement: Supplementary file 2 — Bespoke user-tested patient information sheet. (PDF 328 kb) [file 13063_2017_1797_MOESM2_ESM.pdf]

What if there is a problem?

If you are concerned about any aspect of the study, please contact the REFORM study co-ordinator, Mrs Sarah Cockayne, who will do her best to answer your questions. Please see section 7 below for her contact details.

If you prefer you can contact the local REFORM study Principal Investigator, < INSERT PI NAME > or the NHS Patient Experience team < INSERT NHS PATIENT EXPERIENCE TEAM/PALS DETAILS >.

We do not expect any harm or distress to happen as a result of this study, but there are no special compensation arrangements. If you are harmed due to negligence, then you have grounds for legal action but you may have to pay for it. Regardless of this if you wish to complain, or have any concerns about any aspect of the way you have been approached or treated during the study, the normal NHS complaints mechanisms are available to you.

7 Contact details

If you want any further information, please contact us. A friend or relative may speak to us on your behalf if you wish.

The REFORM study has a website at

www.york.ac.uk/healthsciences/trials-unit/reform/study

REFORM study coordinator:

Mrs Sarah Cockayne
York Trials Unit
ARRC Building
University of York
York YO10 5DD
Tel: **01904 321736**

Podiatry contact:

< INSERT PI NAME >
< INSERT PI ADDRESS >
< INSERT PI TEL NO >

8 What do I do now?

Having read the information, you may

- want to take part, or
- not want to take part, or
- be unsure and want more information.

Yes, I want to take part

If you want to take part in the study, please fill in and sign the **yellow** consent form and the background information form and return them in the envelope provided (no stamp needed). Please keep this information booklet.

If you need any help to complete the forms, please phone us and we will be happy to help.

We will then write to you again in a few weeks to ask you to fill in some short questionnaires. We will write to you to confirm that you are involved in the study and tell your GP.

No, I do not want to take part

There is no need to do anything, if you do not want to take part.

I am unsure and want more information

We would be very pleased to answer any questions. Please contact the study coordinator, Mrs Sarah Cockayne, (01904 321736) or the local podiatrist and REFORM study Principal Investigator, < INSERT PI NAME >. If there is no answer, please leave a message and we will contact you as soon as possible.

If you would like to talk to someone else for general information and advice about taking part in research, contact the NHS Patient Experience team < INSERT NHS PATIENT EXPERIENCE TEAM/ PALS DETAILS >.

Thank you for reading this information booklet.

The REFORM study: a research study about falls. Can you help?

We invite you to take part in a study

- Before you decide whether to take part, it is important that you understand why this study is being done and what it will involve.
- Please take time to read this booklet carefully. Discuss it with others if you wish. Take time to decide whether or not you want to take part.
- It is up to you to decide whether or not to take part. Saying no will not affect the podiatry care you receive.
- Please contact us if you would like to ask any questions.
- Thank you for reading about the study.

The REFORM study: the essentials

- We want to find out how to reduce the number of falls that older people have.
- The study is being run by the University of York. It is funded by the NHS.
- We are inviting all people aged 70 and over who attend the podiatry clinic, whether or not they have ever fallen.
- In the study some people will receive normal podiatry care. Others will receive extra podiatry care, including insoles. These people will be selected by chance, like tossing a coin.
- If you take part, we will ask you to fill in some questionnaires over the coming months.

What do I do now?

If you want to take part, fill in the **yellow** consent form and short background information form and post them to us in the envelope provided.

Please turn over to read the full information about the study

THE UNIVERSITY *of York*

< INSERT LOCAL TRUST LOGO >

REFORM

Contents

1 Why are we doing this study?
2 Why am I being asked to take part?
3 What do I need to know about the podiatry care in this study?
4 What will I need to do if I take part?
5 Possible benefits and disadvantages of taking part.
6 More information about taking part.
7 Contact details
8 What do I do now?

How to contact us

If you have any questions about this study, please contact:

Mrs Sarah Cockayne
York Trials Unit
ARRC Building
University of York
York YO10 5DD
Tel: **01904 321736**

1 Why are we doing this study?

Falling is a common problem among older people. In fact, up to half of people aged over 80 may fall each year. People may fall for a number of reasons, including problems with their balance or eyesight. Unfortunately, some falls cause serious injury, such as a broken bone.

People often think that falls are a result of ageing and that little can be done to stop them. Although it may not be possible to stop all falls, there are many ways to reduce the numbers of falls someone has. These include doing exercises, or checking their eyesight or the medicines they take.

In this study, called the REFORM study, we want to find out how likely older people are to fall and find ways to reduce the number of falls. For example, we would like to know if foot and ankle exercises or wearing insoles prevent falls. The REFORM study is looking at these questions in people aged 70 or over.

2 Why am I being asked to take part?

All people aged 70 or over who have attended the podiatry clinic at White Cross Court, York are being invited to take part. We hope that about 1,700 people in total will agree to take part in this study from clinics across the UK.

Because this information has been sent to you by the podiatry clinic, your name and address is not available to the University researchers unless you choose to give it to them by agreeing to take part.

It is entirely up to you whether or not you decide to take part. Taking part is voluntary.

If you would like more information about the study, please contact us. The details are on page 4 of the booklet.

3 What do I need to know about the podiatry care in this study?

The study is looking at two different forms of podiatry care. Some people in the study will have:

A. Extra podiatry care,

and others will have

B. Normal care from your GP and podiatrist.

It is not possible to offer everyone the extra podiatry care, so these people will be selected by chance, like tossing a coin.

A. Extra podiatry care

If you are offered extra podiatry care we will send you some advice on how to prevent falls, then ask you to see a podiatrist twice at your podiatry clinic. The first appointment will last an hour and the second appointment 20 minutes. Your podiatrist will contact you to make appointments at times that are best for you.

- At the first appointment, the podiatrist will assess your outdoor shoes and advise on your footwear. They will do any routine podiatry care as needed.

  They will also measure your feet and fit an orthotic device (a type of insole for your shoe). They will show you foot and ankle exercises to do at home 3 times a week.
- At the second appointment, the podiatrist will check the fitting of your insoles, and see how you are getting on with the foot and ankle exercises.

We will post you some exercise diaries to fill in, at 1, 3, 6 and 12 months after the start of the study.

B. Normal care from your GP and podiatrist.

If you are offered normal care from your GP and podiatrist, your GP care will continue as usual.

- If you are already having podiatry treatment not related to the study, you will continue with that as usual.
- If you are not currently having podiatry treatment, you will not be asked to attend the clinic.
- We will send you some advice on how to prevent falls.

4 What will I need to do if I take part?

If you agreed to take part, we would first ask you to fill in some short questionnaires about yourself and return them to us.

We would then send you a calendar, which we would ask you to complete and return to us each month. It records whether or not you have fallen during that month.

After 6 and 12 months, we will send you some questionnaires to fill in. We expect that they will take you about 20 minutes to fill in on each occasion.

If you do have a fall at any time, we would ask you to ring the researchers at the University of York, who will ask you some questions about it.

We would ask all people in the study (whether receiving extra podiatry care or normal care) to fill in and return the questionnaires that we will post out.

We will provide envelopes and no stamps are needed.

If you agreed to take part in the study, we would ask for your help for no more than 2 years in total. The information from you will allow us to follow your health and to see if it changes over time. It will let us look at the benefit of podiatry care.

5 Possible benefits and disadvantages of taking part

We cannot promise that taking part will help you personally. However, the study may help us to find out how we can improve balance and reduce the number of falls that older people have.

Taking part will involve some of your time to fill in questionnaires. If you are offered the extra podiatry care, you will be asked to attend the podiatry clinic on two occasions. Unfortunately, we cannot offer any expenses or payments to people who take part in the study. We cannot think of any other disadvantages.

6 More information about taking part

Changing your mind

If you decide to take part, you can still change your mind at any time, without giving a reason. Saying no to the study, or taking part and later changing your mind, will not affect the care you receive at the clinic.

Your GP

If you agree to take part, we will inform your GP. We will also contact your GP if we have any concerns about your health during the study. If you do have a fall, we will ask you if we can contact your GP to find out more information.

Who is running and funding the study?

This study is being run by the University of York.

The research has been funded by the NHS National Institute of Health Research, Health Technology Assessment programme (NIHR HTA).

This study is due to finish in 2015. All patients who take part will be sent a summary of the results. You can also request copies of any published data by contacting the study coordinator.

If you decide not to take part in the study but would like to receive a copy of the results, you can contact the Trials Unit directly, at the University of York (see page 4).

All research in the NHS is looked at by an independent body, called a Research Ethics Committee, to protect your safety, rights, well-being and dignity. This research has been reviewed and approved by the East of England (Cambridge East) NHS Research Ethics Committee.

Other studies

If you agree to take part in this study, we may ask you to join other studies on balance and reducing falls, being carried out by researchers in the REFORM team.

You do not have to take part in any other study, and you would be sent more information about them before you decide. If you do agree to us contacting you about other studies we will keep all personal and anonymised data for a total of 5 years, to allow us to do this.

Confidentiality

Any information you provide to us in the REFORM study will be treated in confidence.

When you join the study we will record your name, address, phone number and date of birth and keep a copy of your signed consent form. This information will be stored securely at the University of York (under the Data Protection Act, 1998).

When we publish the results of the study, your name will not be mentioned and we will ensure that no-one taking part can be identified from the study results. If you decide to stop taking part in the study, the information you had provided up to that point would be used.

If you agreed to take part in the study, members of the REFORM research team at the University of York may look at your medical records. This is only to collect information for the study. People from the NHS Trust where the study is being conducted, may also look at your records to check that the study is being carried out correctly.
