# Supplementary material for: An optimised patient information sheet did not significantly increase recruitment or retention in a falls prevention study: an embedded randomised recruitment trial
Source: Trials. 2017 Mar 28;18:144. doi: 10.1186/s13063-017-1797-7 (PMC5370466; doi:10.1186/s13063-017-1797-7)
Supplement: Supplementary file 3 — Template-developed patient information sheet. (PDF 162 kb) [file 13063_2017_1797_MOESM3_ESM.pdf]

1 Why are we doing this study?

Falling is a common problem in people over 65. About half of people over 80 will fall each year. People fall for many reasons. This may be due to them having problems with their balance or their eyesight. Some people hurt themselves when they fall over. Some may break a bone.

Preventing falls

People often think that falling over is part of getting older and that you can't stop it from happening. However, this is not true. Although it may not be possible to stop every fall, it may be possible to reduce the number you have by:

- doing exercises,
- having your eyes tested,
- getting the doctor to look at the medicines you're taking.

What will happen in this study?

In this study we are looking at the health of people over the age of 70 and the likelihood of them having a fall. We want to find out if improved podiatry care can help stop people falling over. If you are interested in having extra podiatry care, for example doing foot and ankle exercises, then you may be offered this as part of the study at a later date.

The study will take us three and a half years to do. However, we will only ask for your help for a maximum of 24 months.

2 Why am I being asked to take part?

Your podiatry clinic is taking part in this study.

- The podiatry clinic is sending this information sheet to all patients aged over 70 who have attended their clinic.
- We hope that 1700 people will take part in the study.
- Because your podiatry clinic has sent this information to you, your name and address is not available to the researchers unless you choose to give it to them by agreeing to take part in the study.

We would like you to read this information sheet to see if you want to take part.

If you would like to take part in the study

- Please fill in and sign the yellow 'consent' form and 'background information' form.
- Return them in the prepaid envelope provided.
- We will post a copy of your signed consent form back to you.

3 What will happen if I take part in the study?

If you agree to take part the care you get from your GP and podiatrist will continue as normal. We will give you a leaflet telling you what things you can do to stop having a fall. Some people may be offered some extra podiatry visits at a later date to look at ways of reducing falls. It will not be possible to offer everyone some extra podiatry visits, so these people will be selected by a computer which has no information about the individual i.e. by chance.

What will happen if I am offered the extra podiatry visits?

The podiatrist will contact you and make two appointments for you to come to the local podiatry clinic.

The first appointment will last about an hour. The podiatrist will

- Assess your outdoor shoes.
- Give you advice about your footwear.
- Give you an insole to wear in your shoe called an orthotic.
- Show you some foot and ankle exercise, to do at home 3 times a week.
- Arrange any routine podiatry care you need.

The second appointment will last about 20 minutes. The podiatrist will check if any changes need to be made to the insole and check how you are getting on with the foot and ankle exercises.

Collecting information in the trial

Everyone will be asked to

- Fill in a monthly falls calendar,
- Fill in some questionnaires after 6 and 12 months.

This information will let us follow your health and see if it changes over time. It will also contribute to the wider study we are carrying out about the health of people over 70 and the benefits of long term podiatry treatments.

If you are offered extra podiatry visits we will send you some extra questionnaires. This is to find out how often you are wearing your insole and doing your exercises. We will send these to you after 1, 3, 6 and 12 months.

The questionnaires will take about 20 minutes to fill in.

If you fall during the study and if you agree:

- We would like you to ring us. We will then ask you some questions about your fall.
- We may ask your GP for some information.
- We may look at your medical records so that we can check the data.

We may ask you to have an interview with one of our research team. This will be to talk about how to improve people's balance and reduce the number of falls they have.

4 Possible advantages and disadvantages of taking part

We cannot promise that taking part in this study will help you. However, the results of the study may help us find out how we can improve people's balance and stop them from falling.

Taking part will involve some of your time to fill in the questionnaires. If you are offered extra podiatry care you will need to go to the podiatry clinic for two additional appointments.

5 More information about taking part

Do I have to take part?

No, it is up to you to decide whether or not to take part. If you decide you do not wish to take part in the study, you **do not** have to return any forms to us. If you would like more information then please get in touch with us. Our details are at the end of this leaflet.

What if I change my mind?

You can stop taking part in the study at any time. You do not have to give a reason. Deciding to stop at any time, or not to take part, will not affect any care you get from your podiatrist or doctor.

What if there is a problem?

If you are worried or have a problem about any part of this study, phone the study co-ordinator – their details are at the end of this leaflet. They will do their best to answer your questions.

If you don't want to speak to the study co-ordinator you can talk to:

- The local principal investigator Mr Tony Carter, tel: 01904 724222, or
- Your local Patient Advice and Liaison Service (PALS), tel: 01423 555499, email: thepatientexperienceteam@hdft.nhs.uk

We do not expect any harm or distress to happen as a result of this study. However, there are no special compensation arrangements. If you are harmed because of the fault of someone, you are able to take legal action. However, you may have to pay for it.

What if I want to complain?

If you wish to complain, or have any concerns about the way you have been approached or treated, the normal NHS complaints mechanisms are available to you.

What if I have a health problem?

If we have any concerns about your health during the study, if you agree, we will talk to your GP.

What will happen to the information collected about me?

Any information you give us will be kept confidential. The University of York will keep a record of your name, address, telephone number, email address, date of birth and a copy of your signed consent form.

We will store all information securely, which means that only people connected with the study can see it.

People from the regulatory authorities such as the NHS Trust where the study is being run may look at your records to check that the study is being carried out correctly.

If you no longer want to take part in the study, unless you tell us otherwise, we will use the information you have already given us. But we will make sure that you cannot be identified.

The results will be published in a report. Your name will not be mentioned in any publications and we will make sure you cannot be identified from any details.

Will my GP be involved?

If you agree we will let your GP know that you are taking part in this study. If you have a fall, we may contact your GP to find out some further information.

Please turn over

Expenses and payments

Unfortunately we cannot give any direct expenses or payments to people who take part in the study.

Will you ask me to take part in any other studies?

If you agree to take part in this study, we may ask you to take part in other studies about improving balance and reducing falls. These are being done by researchers in our team. You do not have to take part in any of these other studies. We will send you more information about them before you need to decide.

If you agree to us contacting you again we will keep your personal details and anonymised data for 5 years so that we can send you some more information about the other studies before you need to decide.

Who is organising and funding this study?

This study is organised by the University of York. It is funded by the Department of Health (as part of the 'National Institute of Health Research' Health Technology Assessment programme).

All research in the NHS is looked at by an independent group of people, called a Research Ethics Committee. They are there to protect your safety, rights, well-being and dignity. This research has been reviewed and approved by East of England – Cambridge East NHS Research Ethics Committee.

How can I find out about the results of the study?

This study is due to finish in the summer of 2015. All patients who take part will be sent a summary of the results. People who decide not to take part can also get a summary by contacting the study coordinator (see contact information details at the end of this leaflet).

6 What do I have to do now?

If you would like to take part in the study

- Please fill in and sign the yellow 'consent' form and 'background information' form.
- Return them in the prepaid envelope provided.
- Please keep this information sheet.
- We will post a copy of your signed consent form back to you.

If you need any help with filling in the forms, please phone us and we will be happy to help.

We will write to you a few weeks later to ask you to complete a questionnaire. We will also let your GP know you're taking part in the study.

I'm not sure about taking part – where can I get more information about the study?

We would be very pleased to answer any questions you may have. Please contact the study co-ordinator, Mrs Sarah Cockayne, tel: 01904 321736 or your local podiatrist Tony Carter, tel: 01904 724222.

7 Contacts for more information

If you need any further information, please contact us.

A friend or relative may speak to us on your behalf if you wish. There is a 24 hour answering machine – please leave a message and one of the research team will contact you as soon as possible. The REFORM study also has a website at www.york.ac.uk/healthsciences/trials-unit/reform/

Contact details

Study coordinator: Mrs Sarah Cockayne

Telephone: 01904 321736

Address: York Trials Unit, Lower Ground Floor, ARRC Building, University of York, Heslington, York YO10 5DD

Is there anyone else I can talk to about the study?

- For further information about the study please contact your local podiatrist Mr Tony Carter, telephone 01904 724222, Podiatry Department, White Cross Court, White Cross Gardens, Huntington Road, York, YO31 8FT
- For further general information about research please contact your local Patient Advice and Liaison Service (PALS) Email: thepatientexperienceteam@hdft.nhs.uk or telephone 01423 555499.

Thank you for reading this information sheet.

Podiatry care and preventing falls in the over 70s - a research study

REFORM

We invite you to take part in a research study

- Before you decide whether to take part, it is important for you to understand why the research is being done and what it will involve.
- Please take time to read the following information carefully. Discuss it with your friends and family if you wish. Take time to decide whether or not you wish to take part.
- You are free to decide whether or not to take part. If you choose not to take part, this will not affect any care you may get from your own podiatrist or doctor.
- Ask us if there is anything that is not clear, or if you would like more information. Also ask us if you would like help with filling in the forms.
- Thank you for reading this information. If you decide to take part please keep this information sheet.

Important things you need to know about the study

- This study is looking at ways to reduce the number of falls older people have.
- People aged over 70 who have visited their podiatry clinic are being sent this information.
- People will be asked to fill in some questionnaires and monthly falls calendars. They may also be asked to take part in an interview.
- Some patients may be offered extra podiatry visits at a later date.
- If you want to take part, fill in the yellow 'consent' form and the 'background information' form and send them back to us.
- You can stop taking part in the study at any time. You do not have to give a reason.

Please turn over to read the full information about the study

THE UNIVERSITY of York

Contents

1 Why are we doing this study?
2 Why am I being asked to take part?
3 What will happen if I take part in the study?
4 Possible advantages and disadvantages of taking part
5 More information about taking part
6 What do I have to do now?
7 Contacts for more information

How to contact us

If you have any questions about this study, please talk to the study coordinator at:

York Trials Unit
University of York
Ground Floor
ARRC Building
York YO10 5DD
Tel: **01904 321736**
